# The Dutch Healthy Diet index as assessed by 24 h recalls and FFQ: associations with biomarkers from a cross-sectional study

**DOI:** 10.1017/jns.2013.28

**Published:** 2014-01-02

**Authors:** Linde van Lee, Edith J. M. Feskens, Eveline J. C. Hooft van Huysduynen, Jeanne H. M. de Vries, Pieter van 't Veer, Anouk Geelen

**Affiliations:** Division of Human Nutrition, Wageningen University, PO Box 8129, 6700 EV Wageningen, The Netherlands

**Keywords:** Dutch Healthy Diet index, Dietary patterns, Biomarkers, Dietary assessment methods, 24hR, 24 h recall, ADF, acidic drink and food, DHD-index, Dutch Healthy Diet index, DNFCS-2003, Dutch National Food Consumption Survey of 2003, EFCOVAL, European Food Consumption Validation, TFA, *trans*-fatty acid

## Abstract

The Dutch Healthy Diet index (DHD-index) was developed using data from two 24 h recalls (24hR) and appeared useful to evaluate diet quality in Dutch adults. As many epidemiologic studies use FFQ, we now estimated the DHD-index score using FFQ data. We compared whether this score showed similar associations with participants' characteristics, micronutrient intakes, and biomarkers of intake and metabolism compared with the DHD-index using 24hR data. Data of 121 Dutch participants of the European Food Consumption Validation study were used. Dietary intake was assessed by two 24hR and a 180-item FFQ. Biomarkers measured were serum total cholesterol and carotenoids, EPA + DHA in plasma phospholipids and 24 h urinary Na. A correlation of 0·48 (95 % CI 0·33, 0·61) was observed between the DHD-index score based on 24hR data and on FFQ data. Classification of participants into the same tertiles of the DHD-index was achieved for 57 %. Women showed higher DHD-index scores. Energy intake was inversely associated with both DHD-index scores. Furthermore, age and intakes of folate, Fe, Mg, K, vitamin B_6_ and vitamin C were positively associated with both DHD-index scores. DHD-index scores showed acceptable correlations with the four combined biomarkers taking energy intake into account (*r*_24hR_ 0.55; *r*_FFQ_ 0.51). In conclusion, the DHD-index score based on FFQ data shows similar associations with participants' characteristics, energy intake, micronutrient intake and biomarkers compared with the score based on 24hR data. Furthermore, ranking of participants was acceptable for both methods. FFQ data may therefore be used to assess diet quality using the DHD-index in Dutch populations.

Nutrients and single foods have been used in many epidemiological studies as dietary exposures to examine associations with various disease outcomes. To better reflect the complexity of dietary intake, an alternative approach is to investigate overall diet quality. This can be assessed through diet indices, which may give insight into the association of foods, combinations of nutrients and other dietary components with health outcomes^(^^1^^–^^5^^)^.

We recently developed the Dutch Healthy Diet index (DHD-index) that consists of ten components representing the Dutch Guidelines for a Healthy Diet of 2006^(^^6^^)^. In that study, we used data from the Dutch National Food Consumption Survey of 2003 (DNFCS-2003) to examine the association of the DHD-index with energy and micronutrient intakes. We found an inverse association with energy intake and positive associations with several micronutrients when adjusted for energy intake. We concluded that the DHD-index can be used to estimate adherence to the Dutch dietary guidelines and as a monitoring tool in public health research^(^^6^^)^. In the DNFCS-2003, two non-consecutive 24 h recalls (24hR) were used to assess dietary intake. In many epidemiological studies, however, a FFQ is used instead^(^^7^^)^. To evaluate wider applicability of the DHD-index, it is important to compare the DHD-index based on FFQ data with the index based on 24hR data.

A FFQ is designed to assess usual intake whereas 24hR assess detailed information on dietary intake of 1 d or more. Due to natural day-to-day variation within an individual, comparing the DHD-index score based on FFQ data is expected to differ from the DHD-index score based on 24hR. For example, as fish is considered episodically consumed in the Netherlands, estimations of fish intake from a FFQ are expected to be higher compared with data from two 24hR. This example of measurement error is a feature of the dietary assessment method as such and will influence the DHD-index scores. Therefore, it is important to not only compare the DHD-index based on FFQ data with the DHD-index based on 24hR data, but also examine associations with objective urinary and plasma biomarkers of dietary intake and metabolism. Serum total cholesterol, EPA, DHA and several carotenoids have shown significant associations with existing indices of diet quality^(^^8^^–^^12^^)^. These significant correlations between diet quality indices and single biomarkers ranged between 0.19 and 0.44^(^^8^^,^^11^^,^^12^^)^.

Our objective was to assess whether the DHD-index score based on FFQ data showed similar associations with participants' characteristics, micronutrient intakes, and biomarkers of dietary intake and metabolism compared with the DHD-index score based on 24hR data. Furthermore, we will compare the ranking of participants between the DHD-index scores based on the two dietary assessment methods. The biomarkers of dietary intake were selected based on the literature^(^^8^^–^^12^^)^ and on the availability of data.

## Methods

### Subjects

Data of the Dutch participants of the European Food Consumption Validation (EFCOVAL) study, including 121 men and women aged 45–65 years, were used for the present study. All subjects were healthy individuals representing all educational levels. Subjects were excluded if they could not speak and write Dutch, were currently taking diuretics, were pregnant or lactating, had diabetes mellitus or kidney disease, and had been donating blood or plasma less than 4 weeks before the study. All subjects signed an informed consent and the present study was conducted according to the guidelines laid down in the Declaration of Helsinki. All procedures involving human subjects were approved by the medical ethical committee in Wageningen.

### Study design

The EFCOVAL study is an observational study in five European countries and has been described in more detail by Crispim *et al.*^(^^13^^,^^14^^)^. The aim of the study was to validate the duplicate 24hR method using EPIC-Soft (International Agency of Research on Cancer); a computerised 24hR program that follows standardised procedures^(^^15^^,^^16^^)^.

At enrolment, all subjects filled in the Short Questionnaire Assessing Health Enhancing Physical Activity (SQUASH)^(^^17^^)^ and a general questionnaire on lifestyle, food habits and supplement use. Fish oil supplement users were identified when at least on one of the recalled days or during the past 3 months at least one supplement containing EPA or DHA was consumed. Furthermore, body weight and height were measured following standardised protocols at the study centre. After that, a 24hR and a 24 h urine collection were obtained covering the same reference day. The second 24hR and urine collection were obtained at least 1 month after the first one. At the end of the study period, all subjects received a FFQ by mail and filled it in at home.

### Dietary assessment methods

Two non-consecutive 24hR were collected per subject, one by phone and one face to face at the research centre. All days of the week and the two modes of administration of 24hR were randomised among subjects, whereas the intake on Saturdays was recalled 2 d later on Mondays. Interviewers were all trained in interviewing techniques and in using EPIC-Soft (version 9.16). Portion size estimation was done using household measures, weight/volume, standard units and portions, bread shapes and photographs. Nutrient intakes were calculated using the Dutch food composition table^(^^18^^)^.

The 180-item semi-quantitative FFQ was developed to assess intake of energy, macronutrients, dietary fibre and selected vitamins^(^^19^^)^. All questionnaires were checked on unusual or missing values, and, if necessary, subjects received a telephone call to obtain additional information. Average daily nutrient intakes were calculated by multiplying frequency of consumption of food items by portion size and nutrient content per g based on the Dutch food composition table^(^^18^^)^.

### Biomarkers

More detail on the 24 h urine collections, venepuncture, analyses and storage have been described elsewhere^(^^13^^,^^14^^,^^20^^)^. Briefly, *para*-aminobenzoic acid (PABA) was used to verify completeness of 24 h urine collections. Subjects were asked to fill in a short diary about time of taking PABA, completeness of the urine collection and medication use. Five urine samples with PABA recoveries below 50 % were excluded from the data analyses. Recoveries between 50 and 85 % were proportionally adjusted to 93 % of PABA recovery, as suggested by Johansson *et al.*^(^^21^^)^. Recoveries above 85 % were included without adjustments. Urinary Na was measured by an ion-selective electrode on a Beckman Synchron LX20 analyser (Beckman Coulter) as the biomarker for dietary Na intake^(^^22^^)^.

Non-fasting blood samples were taken by a trained laboratory technician. The percentage of EPA and DHA in relation to the total measured fatty acids (thirty-five fatty acids) was used as the concentration biomarker of fish intake^(^^23^^)^. The carotenoids α-carotene, β-cryptoxanthin, β-carotene, lutein and zeaxanthin were analysed as described by Nguyen *et al.*^(^^24^^)^ and their sum was used as the marker of fruit and vegetable intake^(^^25^^)^. Serum total cholesterol was measured spectrophotometrically on a Synchron LX20 clinical analyser (Beckman Coulter) and was used as the biomarker of SFA and *trans*-fatty acids (TFA)^(^^23^^)^.

### Dutch Healthy Diet index

The DHD-index consists of ten components (physical activity, vegetables, fruit, fibre, fish, SFA, TFA, consumption occasions of acidic drink and food (ADF), Na and alcohol), representing the ten Dutch Guidelines for a Healthy Diet of 2006 (Table 1). The maximum score of each component is 10, resulting in a total score ranging from zero (no adherence) to 100 (complete adherence). The criteria used to calculate the DHD-index have been described in detail elsewhere^(^^6^^)^. Briefly, the required amount of consumption or physical activities stated in the Dutch Guidelines for a Healthy Diet were used as cut-off values for the maximum number of points. For the components physical activity, fruit, vegetables, fish and fibre the minimum score of zero was assigned when no intake or activities were undertaken. For the components SFA, TFA, ADF occasions and Na the minimum score was based on the 85th percentile of the 2 d average intake of a Dutch reference population^(^^26^^)^. These threshold values are recommended for all future use of the DHD-index to make it possible to compare results between different study populations. The cut-off value for the component TFA was lower than the dietary recommendation; consequently the component TFA was scored dichotomously. The cut-off values for the component Na were lowered by 30 % to adjust for Na added during cooking and at the table^(^^27^^,^^28^^)^, which is not taken into account by both dietary assessment methods. The minimum score for the component alcohol was based on the cut-off values of binge-drinking^(^^29^^)^. Between zero and 10 points the score was calculated proportionally.

**Table 1.** Components of the Dutch Healthy Diet index and their cut-off values (maximum score) and threshold values (minimum score)

Component	Minimum score (=0)	Maximum score (=10)
1. Physical activity (per week)*	0 activities	≥5 activities
2. Vegetables (per d)	0 g	≥200 g
3. Fruit + fruit juices (per d)†	0 g	≥200 g
4. Fibre (per d)	0 g/4·2 MJ	≥14 g/4·2 MJ
5. Fish (per d)‡	0 mg EPA + DHA	≥450 mg EPA + DHA
6. SFA (per d)	≥15 en %	<10 en %
7. TFA (per d)	≥1 en %	<1 en %
8. ADF occasions (per d)§	>7 occasions	≤7 occasions
9. Na (per d)	≥2520 mg	<1680 mg
10. Alcohol (per d)	Male: ≥6 drinks	Male: ≤2 drinks
	Female: ≥4 drinks	Female: ≤1 drinks

en %, Percentage energy; TFA, *trans*-fatty acids; ADF, acidic drink and food.

*Activities were at least moderately intensive and minimally 30 min.

† A maximum of 100 g of fruit juice containing vitamin C and folate could be included.

‡ Fish intake was estimated based on dietary fish fatty acids (EPA + DHA) and fish oil capsules.

§ The number of ADF consumption occasions was defined as the number of hours where at least one food or drink with a pH <5·5 and total acidity >0·5 % was consumed.

For the 24hR data, component scores were based on reported 2 d average intake. For calculation with the FFQ data, scores were based on the reported usual intake. The component ADF could not be estimated with FFQ data, as the number of consumption occasions per d was not assessed. Therefore, component ADF occasions were omitted from the index in all further analyses. In addition, the component physical activity was omitted from further analyses because the SQUASH was assessed only once; consequently the component score was the same for both indices based on the two different dietary assessment methods.

### Statistical analyses

Ranking of the participants between the DHD-index scores based on FFQ data and the DHD-index score based on 24hR data was studied by analysing the correlations and cross-classification of tertiles. Partial correlation coefficients were calculated between the DHD-index score and its components based on FFQ data and the DHD-index score based on 24hR data adjusting for energy intake assessed by FFQ and by 24hR. Additional adjustment for sex did not alter the results. Pearson correlations were used for normally distributed variables and Spearman correlations for skewed variables. The 95 % CI of the correlation coefficients were calculated by Fisher's *Z*-transformation. Differences between medians were tested with the Wilcoxon signed-rank test and by the χ^2^ test for the dichotomous TFA component. To study the association of the DHD-index with participants' characteristics and micronutrient intakes, the DHD-index was divided into sex-specific tertiles. Means, standard deviations and *P* for trend were calculated with general linear models.

The four biomarkers were used as independent variables in a linear regression to provide, hypothetically, the best objective ‘marker’ of diet quality based on available data. We expected correlations of 0·4 between the DHD-index and the four linear combinations of biomarkers based on published correlations between single biomarkers and diet indices^(^^7^^,^^10^^,^^11^^)^. The square root of *R*^2^ from linear regression models including energy intake as an independent variable was used to calculate the energy-adjusted correlation coefficient between the DHD-index score and the four biomarkers. The 95 % CI for this correlation was estimated with bootstrap analyses using 10 000 replications. Partial correlation coefficients for the separate biomarkers were calculated for the DHD-index scores based on the two dietary assessment methods and for the component scores of interest adjusting for energy intake. Additional adjustment for sex did not change the results. All statistical analyses were performed using SAS 9·2 (SAS Institute Inc.).

## Results

The mean age of the study population was 56·2 (sd 5·1) years and mean BMI was 26.0 (sd 4·5) kg/m^2^. Almost 50 % of the study population completed a level of higher education and 10 % of the study population followed a diet regimen.

The mean DHD-index score based on FFQ data was 6·0 points higher for women than for men (*P* = 0·003), and 5·7 (*P* = 0·018) points higher for women when the DHD-index was based on 24hR data. The mean DHD-index score for the sum of eight components was 49·9 (sd 13·5) based on 24hR data and 56.0 (sd 11·0) based on FFQ data (*P* < 0·001; Table 2). The median component score for vegetables based on 24hR data was higher than the median based on FFQ data (*P* < 0·001). The four components fibre, fish, SFA and Na showed significantly lower median scores when the scores were based on 24hR data compared with FFQ data. The components fruit, TFA and alcohol showed similar medians for both methods, whereas the alcohol component score distributions were different (*P* < 0·001).

**Table 2.** Dutch Healthy Diet index (DHD-index) and its component scores based on two 24 h recalls (24hR) and on a FFQ in 121 Dutch subjects of the European Food Consumption Validation study and associations between the two scores (Medians and interquartile ranges (IQR), and partial correlations and 95% confidence intervals)

	24hR		FFQ			
	Median	IQR	Median	IQR	Correlation*	95 % CI
DHD-index†	51·7	20·7	57·30	15·8	0·48	0·33, 0·61
Vegetables	8·8	3·3	6·3	5·2	0·29	0·12, 0·45
Fruit	10·0	3·9	10·0	4·4	0·41	0·25, 0·55
Fibre	7·9	3·1	9·0	2·3	0·58	0·45, 0·69
Fish	0·7	5·3	3·3	3·9	0·33	0·16, 0·48
SFA	4·7	8·6	6·5	5·8	0·43	0·27, 0·57
TFA	10·0	0·0	10·0	0·0	0·16	−0·02, 0·33
Na	1·1	7·9	4·1	9·9	0·30	0·13, 0·46
Alcohol	10·0	4·5	10·0	1·6	0·65	0·54, 0·70

TFA, *trans*-fatty acids.

*Adjusted for energy intakes assessed by the FFQ and 24hR.

† Excluding the components acidic drink and food consumption occasions and physical activity.

The results from cross-classification showed that 57 % were classified in the same tertile and 7 % were classified in the opposite tertile when comparing DHD-index score based on FFQ and 24hR data, with Kendall's τ-b coefficient of 0·47 (95 % CI 0·33, 0·60). The correlation between the DHD-index scores based on FFQ and 24hR data was 0·58 (95 % CI 0·45, 0·69) and after energy adjustment this correlation decreased to 0.48 (95 % CI 0·33, 0·61; Table 2). The correlations between the component scores based on 24hR and FFQ data ranged between 0·16 and 0·65. The lowest correlation was observed for the component TFA and was not significant. The two highest correlations were observed for the components alcohol and fibre.

Moderate correlations between the components fruit and vegetables with fibre (*r* 0·42 and *r* 0·46, respectively) and for SFA with TFA (*r* 0·39) were observed when the DHD-index was based on FFQ data.

The participants' age showed a positive trend across the sex-specific tertiles of the DHD-index score based on FFQ data (*P* for trend = 0·004; Table 3). Energy intake showed an inverse trend across the tertiles of the FFQ DHD-index score (*P* for trend <0·0001), while BMI, supplement use, smoking and educational level did not show a significant trend across the tertiles. Intakes of the micronutrients folate, Fe, Mg, thiamin, vitamin B_6_ and vitamin C expressed per 4·2 MJ were positively associated with the DHD-index score based on FFQ data. Intakes of the micronutrients Ca, riboflavin, vitamin A, vitamin B_12_ and vitamin E showed no significant trend across tertiles of the FFQ DHD-index score. The DHD-index score based on 24hR data showed similar positive associations with participants' characteristics and micronutrient intakes. Additionally, vitamin E was positively associated (*P* < 0·022) with the DHD-index score based on 24hR data (data not shown).

**Table 3.** Participants' characteristics, biomarkers and micronutrient intakes across sex-specific tertiles (T) of the Dutch Healthy Diet index (DHD-index) based on FFQ data in 121 Dutch subjects of the European Food Consumption Validation study (Mean values and standard deviations)

	Sex-specific tertiles of the DHD-index*†						
	T1 (*n* 40)		T2 (*n* 41)		T3 (*n* 40)		
	Mean	sd	Mean	sd	Mean	sd	*P* for trend
Age (years)	55·0	5·5	55·5	4·9	58·3	4·5	0·004
BMI (kg/m^2^)	26·9	5·1	25·0	2·9	26·2	5·0	0·479
Energy intake (MJ/d)	8·9	2·2	7·8	2·2	6·6	1·6	<0·001
Supplements users (%)	45·0		65·9		50·0		0·653
Smokers (%)	15·0		2·5		5·0		0·088
Diet regimen (%)	12·5		4·5		12·5		1·000
Education (%)							0·578
Low	22·5		19·5		25·0		
Mediate	30·0		26·8		35·0		
High	47·5		53·6		40·0		
Biomarkers‡							
Carotenoids (μg/100 ml)	114·4	89·1	113·8	84·7	128·7	90·2	0·234
Fish fatty acids (% of total fat)	4·5	2·2	4·3	2·2	4·3	2·2	0·019
Total cholesterol (mmol/l)	5·6	2·2	5·5	2·2	5·6	2·2	0·763
Urinary Na (mmol/d)	388·9	1164·9	171·6	1095·6	293·1	1168·2	0·538
Micronutrients (per 4·2 MJ)							
Ca (mg)	545	160	544	157	540	116	0·874
Folate (μg)	90·1	17·1	106·3	27·4	121·1	26·9	<0·001
Fe (mg)	4·8	0·9	5·6	0·9	6·1	1·0	<0·001
Mg (mg)	157·7	25·6	171·1	29·8	186·8	31·7	<0·001
K (mg)	1634	248	1830	246	2028	344	<0·001
Riboflavin (mg)	0·8	0·3	0·8	0·2	0·9	0·2	0·839
Thiamin (mg)	0·7	0·1	0·7	0·1	0·8	0·1	0·004
Vitamin A (RAE)	575	269	591	267	576	274	0·988
Vitamin B_6_ (mg)	0·9	0·2	0·9	0·1	1·0	0·1	<0·001
Vitamin B_12_ (μg)	2·2	0·9	2·1	1·1	2·3	1·1	0·829
Vitamin C (mg)	35·9	17·6	51·1	30·1	64·2	22·3	<0·001
Vitamin E (mg)	5·3	1·4	5·7	1·4	5·7	1·2	0·155

RAE, retinol activity equivalents.

*Excluding the components acidic drink and food consumption occasions and physical activity.

† Cut-off values of tertiles for men: 42.1 and 53.1. Cut-off values of tertiles for women: 47.7 and 61.1.

‡ Adjusted for energy intake.

The correlation, estimated using a linear regression model, between the four biomarkers serum carotemoids, EPA + DHA, total cholesterol and urinary Na on one hand, and the DHD-index score based on 24hR data on the other hand, was 0·55 (95 % CI 0·44, 0·68), and for the DHD-index score based on FFQ data was 0.51 (95 % CI 0·40, 0·67). The DHD-index scores based on FFQ data and 24hR data were positively correlated with serum EPA + DHA (both 0·19; Table 4). No significant correlations were observed between the biomarkers serum carotenoids, urinary Na, or serum total cholesterol and the DHD-index scores based on the two dietary assessment methods.

**Table 4.** Associations between biomarkers and the Dutch Healthy Diet index (DHD-index) and seven separate components of the DHD-index based on FFQ and 24 h recall (24hR) data in 121 Dutch subjects of the European Food Consumption Validation study (Partial correlations* and 95 % confidence intervals)

	Serum carotenoids†		EPA + DHA		Urinary Na		Serum total cholesterol	
	Correlation	95 % CI	Correlation	95 % CI	Correlation	95 % CI	Correlation	95 % CI
DHD-index 24hR‡	0·06	−0·12, 0·24	0·19	0·02, 0·36	−0·04	−0·22, 0·14	−0·04	−0·22, 0·14
DHD-index FFQ‡	0·13	−0·05, 0·30	0·19	0·01, 0·35	0·02	−0·16, 0·20	−0·03	−0·21, 0·15
Vegetables 24hR	0·25	0·07, 0·41						
Vegetables FFQ	0·17	−0·01, 0·34						
Fruit 24hR	0·09	−0·09, 0·27						
Fruit FFQ	0·25	0·08, 0·41						
Fibre 24hR	0·21	0·03, 0·37						
Fibre FFQ	0·20	0·02, 0·37						
Fish 24hR			0·26	0·08, 0·42				
Fish FFQ			0·55	0·41, 0·66				
SFA 24hR							0·00	−0·18, 0·17
SFA FFQ							−0·00	−0·18, 0·18
TFA 24hR							−0·05	−0·22, 0·13
TFA FFQ							−0·03	−0·16, 0·20
Na 24hR					−0·16	−0·33, 0·02		
Na FFQ					−0·23	−0·39, −0·05		

TFA, *trans*-fatty acids.

* Adjusted for energy intake.

† α-Carotene, β-cryptoxanthin, β-carotene, lutein and zeaxanthin.

‡ Excluding the components acidic drink and food consumption occasions and physical activity.

The vegetable component scores based on FFQ data and 24hR data were both positively correlated with serum carotenoids (*r*_24hR_ 0·25 and *r*_FFQ_ 0·17), although the correlation was not significant for the FFQ data (Table 4). For the fruit component score based on FFQ data, a significant correlation was observed with serum carotenoids (*r* 0·25; 95 % CI 0·08, 0·41), while it was 0.09 and non-significant for the fruit component score based on 24hR data. Significant correlations were observed between serum carotenoids and the fibre component score based on FFQ data (*r* 0·20) and the fibre component based on 24hR data (*r* 0·21). Serum EPA + DHA was associated with the fish component scores, the correlation being higher when based on FFQ data compared with 24hR data (*r* 0·53 *v. r* 0·30, respectively). Urinary Na was inversely correlated with the Na component although, not significantly, for the Na component based on 24hR data. These inverse correlations were expected, because higher scores on the component Na were expected to be associated with lower dietary Na intake. No significant associations were observed between total cholesterol and the components SFA and TFA for both dietary assessment methods.

## Discussion

In the present study, we examined the performance of the DHD-index based on a 180-item FFQ by studying its association with participants' characteristics, micronutrient intakes and biomarkers of intake and compared its performance with the performance of the DHD-index based on 24hR data. The DHD-index score based on FFQ data showed similar associations with participants' characteristics and micronutrient intakes as the DHD-index score based on 24hR data. For both dietary assessment methods, correlations between DHD-index and the combined four biomarkers were higher than the expected magnitude of 0·4 based on the literature. These results confirm the previous conclusion that the DHD-index based on 24hR can be used to assess diet quality and suggest that the DHD-index based on FFQ data can also be used to rank participants according to their diet quality in Dutch populations.

In the present study, the component ADF consumption occasions was omitted, because the number of consumption occasions could not be assessed by the FFQ. Previously, the component ADF consumption occasions showed not to be discriminating in ranking subjects according to the guideline^(^^6^^)^. Furthermore, no significant differences were seen in the associations with the DHD-index based on 24hR and participants' characteristics, micronutrients and biomarkers when the component ADF consumption occasions was excluded (data not shown). This suggests that the component ADF consumption occasions may be omitted to arrive at a more simple form of the index. However, the DHD-index has not yet been evaluated by studying diet–disease associations, which might alter this conclusion.

Ranking of the participants based on the two DHD-index scores was studied by examining the correlations and cross-classification. The correlation between the DHD-index based on FFQ data and based on 24hR data was comparable with the correlation (*r* 0·48) reported by Benítez-Arciniega *et al.*^(^^30^^)^, who compared the ‘Modified Mediterranean diet score’ based on FFQ data with the score based on twelve 24hR. However, our observed correlation was lower than the correlation (*r* 0·72) reported by Newby *et al.*^(^^11^^)^. The latter correlation, however, compared the ‘Diet Quality Index Revised’ based on FFQ data with the index based on two 1-week diet records. The reference periods covered by these two dietary assessment methods are probably more comparable with each other than the reference periods covered by our FFQ and two 24hR, which could explain the lower correlation in the present study.

Well over a half of the participants in the present study were classified into the same tertile; this result is similar to the results from cross-classifications between a FFQ and 24hR on food groups^(^^30^^,^^31^^)^. Furthermore, Kendall's τ-b coefficient showed a moderate agreement between the tertiles of the DHD-index based on FFQ and 24hR data. Based on the present results, we can conclude that ranking of participants was acceptable for both DHD-index scores.

The DHD-index component scores based on 24hR data and the DHD-index component scores based on FFQ data were all significantly correlated with each other, except for the component TFA. This might be due to the fact that the component TFA was scored dichotomously and thus showed little variation. The component alcohol showed the highest correlation between 24hR and FFQ; this could be due to the fact that FFQ and 24hR are both known for a satisfactory ranking between individuals according to alcohol intake^(^^32^^)^. The correlation between the vegetable components was rather low. In most validation studies the FFQ tends to overestimate vegetable intake compared with vegetable intake assessed by multiple 24hR^(^^33^^)^; however, the results of the present study showed the opposite. We could not explain this discrepancy. The correlation between Na components was rather low, probably because the FFQ was not specifically designed to assess Na intake levels. Furthermore, fish components were also rather poorly correlated, probably due to the fact that two recalls are unable to assess the usual intake of episodically consumed foods such as fish^(^^34^^)^.

To improve comparability between the two DHD-index scores based on FFQ and 24hR data, usual intakes could be estimated for 24hR data by statistical models, like the National Cancer Institute Method and the multiple source method^(^^34^^,^^35^^)^. These methods eliminate intra-individual variability from the data. Unfortunately, the estimation of usual intakes requires a bigger sample size^(^^36^^)^ and the statistical methods may have their limitations^(^^37^^)^. Age and energy intake showed significant trends across the sex-specific tertiles of both DHD-index scores. The inverse association of the DHD-index score with energy intake was also observed in the population of the DNFCS-2003^(^^6^^)^. The positive association with age, however, was not seen in that population. This may be due to the smaller age range (19–30 years) in the DNFCS-2003 population compared with the age range (45–65 years) of the EFCOVAL study population.

The positive associations of micronutrient intakes with the DHD-index score based on FFQ data in the present study were similar to the associations with the DHD-index based on 24hR and to our earlier findings based on DNFCS-2003 data^(^^6^^)^. Newby *et al.* found similar associations for the ‘Diet Quality Index Revised’ with the micronutrients vitamin A, vitamin B_6_, vitamin C, folate, Mg and Fe^(^^11^^)^. In the present study, however, vitamin E also showed a positive association across tertiles of the DHD-index based on 24hR (*P* < 0·022), which was comparable with others^(^^8^^–^^10^^)^. We assumed that the combination of the four biomarkers was the best available approach to evaluate diet quality as estimated by the DHD-index. The magnitude of the correlations was higher than the expected correlations of 0·4 based on published correlations of diet indices with single biomarkers^(^^8^^,^^11^^,^^12^^)^. Based on these results we may conclude that for both dietary assessment methods the DHD-index can be used to assess diet quality at the population level.

A limitation of both dietary assessment methods is the inaccurate assessment of dietary Na intake. Dietary Na intake assessed by the two methods is probably underestimated due to lacking data on salt added during cooking or at the table^(^^38^^)^. Furthermore, the FFQ used was not specifically designed for the estimation of Na and did not include questions on all Na-rich food products such as soya sauce. By lowering the cut-off values by 30 %, we tried to adjust for these measurement errors. In the present study, however, we also measured urinary Na, the preferred method of estimating dietary Na intake^(^^22^^)^. The mean Na component score was 2·4 (sd 3·5) when based on urinary Na, 3.5 (sd 4·1) when based on 24hR data, and 4·8 (sd 4·3) when based on FFQ data. These differences are quite substantial; consequently, conclusions regarding the DHD-index component score based on Na intake assessed by FFQ or 24hR data must be drawn with caution. Preferably, data of urinary Na are used for estimation of the component Na to overcome measurement errors. If urinary Na is used, the original cut-off values without additional adjustment should be used; maximum points will be assigned when Na intake is lower or equals 2400 mg and zero points will be assigned when Na intake is above 3600 mg.

In the present study, the biomarkers used were initially selected to validate two non-consecutive 24hR using EPIC-Soft within the EFCOVAL study. Unfortunately, the biomarkers carotenoids and total cholesterol have some limitations for the present study. First, the biomarker plasma carotenoids is already known for its modest correlation with fruit and vegetable intake^(^^39^^)^, also observed in the present study. This can be explained by the influence of many other factors such as absorption and metabolism on plasma carotenoid concentrations^(^^40^^)^. Additional adjustment for serum total cholesterol and smoking did not improve the results for plasma carotenoids with the components fruit, vegetables and fibre. Unfortunately, a more accurate biomarker for fruit and vegetable intake is not available.

Second, serum total cholesterol was used as a biomarker for SFA and TFA intake. In the present study, no significant correlations were observed between the DHD-index and serum total cholesterol, which was comparable with the results of others^(^^9^^,^^12^^,^^41^^)^. In some other studies, however, significant associations were observed^(^^8^^,^^11^^,^^42^^)^. Suggested explanations for these discrepancies were the differences between intake levels of populations, the differences between dietary assessment methods, and the differences between indices used^(^^8^^–^^11^^)^. Preferably, serum LDL-cholesterol concentrations should be used to study associations with types of fat intake^(^^43^^,^^44^^)^, but these were not available in the present study.

In conclusion, the DHD-index based on a 180-item FFQ showed similar associations with participants' characteristics, micronutrient intake and biomarkers of dietary intake and metabolism compared with the DHD-index based on two non-consecutive 24hR. Furthermore, the ranking of participants was acceptable for both DHD-index scores. Therefore, both dietary assessment methods can be used to assess diet quality by using the DHD-index in Dutch populations. Future research should focus on the evaluation of the DHD-index by studying associations with disease outcomes.
